# Rifaximin Improves Spatial Learning and Memory Impairment in Rats with Liver Damage-Associated Neuroinflammation

**DOI:** 10.3390/biomedicines10061263

**Published:** 2022-05-28

**Authors:** Paola Leone, Gergana Mincheva, Tiziano Balzano, Michele Malaguarnera, Vicente Felipo, Marta Llansola

**Affiliations:** 1 Mar Lab Department of Neuroscience, NYU Grossman School of Medicine Science Building, New York, NY 10016, USA; paola.leone@nyulangone.org; 2 Laboratory of Neurobiology, Centro de Investigación Príncipe Felipe, 46012 Valencia, Spain; givaylova@cipf.es (G.M.); mllansola@cipf.es (M.L.); 3 Centro Integral de Neurociencias, A.C. HM Hospital Universitario Puerta del Sur CINAC, 28938 Madrid, Spain; tbalzano.hmcinac@hmhospitales.com; 4 Department of Psychobiology, Facultad de Psicología, Universitat de Valencia, 46010 Valencia, Spain; michele.malaguarnera@gmail.com

**Keywords:** hepatic encephalopathy, mild liver damage, rifaximin, microglia, NMDA receptor, spatial learning and memory

## Abstract

Patients with non-alcoholic fatty liver disease (NAFLD) may show mild cognitive impairment. Neuroinflammation in the hippocampus mediates cognitive impairment in rat models of minimal hepatic encephalopathy (MHE). Treatment with rifaximin reverses cognitive impairment in a large proportion of cirrhotic patients with MHE. However, the underlying mechanisms remain unclear. The aims of this work were to assess if rats with mild liver damage, as a model of NAFLD, show neuroinflammation in the hippocampus and impaired cognitive function, if treatment with rifaximin reverses it, and to study the underlying mechanisms. Mild liver damage was induced with carbon-tetrachloride. Infiltration of immune cells, glial activation, and cytokine expression, as well as glutamate receptors expression in the hippocampus and cognitive function were assessed. We assessed the effects of daily treatment with rifaximin on the alterations showed by these rats. Rats with mild liver damage showed hippocampal neuroinflammation, reduced membrane expression of glutamate N-methyl-D-aspartate (NMDA) receptor subunits, and impaired spatial memory. Increased C-C Motif Chemokine Ligand 2 (CCL2), infiltration of monocytes, microglia activation, and increased tumor necrosis factor α (TNFα) were reversed by rifaximin, that normalized NMDA receptor expression and improved spatial memory. Thus, rifaximin reduces neuroinflammation and improves cognitive function in rats with mild liver damage, being a promising therapy for patients with NAFLD showing mild cognitive impairment.

## 1. Introduction

Chronic liver failure can alter brain function and the set of neurological symptoms produced is known as hepatic encephalopathy (HE). Around 40% of cirrhotic patients show minimal hepatic encephalopathy (MHE), MHE induces psychomotor slowing, attention deficits, mild cognitive impairment and motor incoordination [1]. Steatohepatitis, is an early stage of liver disease, characterized by fatty-inflamed liver. Many patients suffering from steatohepatitis may already show mild cognitive impairment before developing cirrhosis [2,3,4]. The prevalence has been reported to be 32% [4].

Studies in animal models show that neuroinflammation is responsible for cognitive and motor impairment in MHE [5,6,7]. Hippocampus modulates spatial learning and memory [8,9] and other cognitive processes such as object recognition memory [10,11] and working memory [12,13,14]. In animal models of MHE, neuroinflammation in the hippocampus (astrocytes and microglia activation and increased levels of pro-inflammatory TNFα and interleukin-1β (IL-1β)) leads to altered glutamatergic neurotransmission, with altered membrane expression of α-amino-3-hydroxy-5-methyl-4-isoxazolepropionic acid (AMPA) and NMDA receptor subunits which is responsible for cognitive impairment [15,16,17,18]. Treatments preventing or reversing neuroinflammation in the hippocampus, such as sulphorafane, sildenafil, extracellular cyclic guanosine monophosphate (cGMP), antagonists of the IL-1 receptor, or infliximab, normalize membrane expression of glutamate receptors and improve spatial learning and memory [15,16,17,18,19,20], supporting that neuroinflammation is a main responsible for cognitive impairment in MHE.

Cirrhotic patients died with HE or MHE also show neuroinflammation [21,22]. Patients who died with steatohepatitis show neuroinflammation in the cerebellum, with infiltration of T lymphocytes and loss of Purkinje and granular neurons [21]. This suggests that also in these patients neuroinflammation would be responsible for cognitive impairment. However, it has not been studied in detail if mild liver failure (steatohepatitis) is enough to induce neuroinflammation in the hippocampus, the characteristics of this neuroinflammation and if this is associated with altered glutamatergic neurotransmission and cognitive impairment. We addressed these questions in the present work.

Neuroinflammation and cognitive impairment in MHE is a consequence of alterations in peripheral inflammation [17,20,23,24]. However, how these peripheral alterations are transmitted into the brain remains unclear. Several mechanisms have been proposed including (1) activation by peripheral pro-inflammatory cytokines (IL-6, IL-17, TNFα) of their receptors in endothelial cells in the blood–brain barrier and transmission of the alterations to neighboring astrocytes in the brain [25,26,27]; (2) infiltration of extracellular vesicles from the blood [28]; and (3) activation by pro-inflammatory cytokines of vagal nerve and transmission of the signals to brain. In some pathologies (multiple sclerosis, cerebral ischemia,…) a main mechanism of transmission of peripheral alterations is by infiltration of peripheral lymphocytes and/or monocytes into the brain [29,30,31,32]. These immune cells are attracted to the brain by release of specific chemokines, such as CCL2. The increase in CCL2 expression in the hippocampus has been associated to recruitment of monocytes, exacerbation of microglia activation, neuronal disfunction, and memory impairment [33,34,35].

We have recently shown that mild liver failure in rats, similar to steatohepatitis in human patients, induces infiltration of lymphocytes and macrophages in the cerebellum [36]. We have also now analyzed infiltration of immune cells in the hippocampus and its possible relationship with neuroinflammation and cognitive impairment.

It has been shown that cognitive impairment may be improved in cirrhotic patients with MHE by treating them with rifaximin, the only treatment approved to prevent appearance of HE [37]. Rifaximin, a semi-synthetic and non-absorbable antibiotic which reduces the risk of overt HE recurrence and mortality rate in comparison to non treated patients [38]. Rifaximin acts on microbiota function [39], has anti-inflammatory effects [40] and improves MHE only in cirrhotic patients which improve peripheral inflammation and immunophenotype [37]. However, how rifaximin affects the brain to improve cognitive function remains unknown. We hypothesized that rifaximin would improve cognitive function in rats with mild liver failure by reducing neuroinflammation and alterations in neurotransmission.

The aims of this work were to assess: (a) if mild liver damage in rats induces hippocampal neuroinflammation; (b) if this is associated with alteration of neurotransmission and cognitive impairment; (c) if infiltration of lymphocytes and/or monocytes may contribute to induction of neuroinflammation; and (d) if treatment with rifaximin reverses the induction of neuroinflammation and of cognitive impairment.

## 2. Materials and Methods

### 2.1. Animal Model and Treatment with Rifaximin

Male Wistar rats (Charles River) weighing 150–180 g were intraperitoneally injected 3 times/week during 4 weeks with 1 mL/kg body weight of carbon tetrachloride (CCl_4_) to induce liver damage. CCl_4_ was prepared 1:10 (*v*:*v*) in corn oil as previously described [36,41] Control rats were intraperitoneally injected with corn oil. Rifaximin (Sigma, St. Louis, MO, USA) was dissolved in 100% ethanol and administered orally (20 mg/kg body weight). Two weeks after first CCl_4_ injection started daily rifaximin treatment and it was maintained until sacrifice. Control rats were orally treated with the same volume of 100% ethanol. At 4 weeks of CCl_4_ injections, this animal model shows liver steatosis, inflammation and fibrosis, but not hyperammonemia [36], similar to mild steatohepatitis in humans. The experiments were approved by the Ethic Committee of Animal Experimentation of our Center and by the Conselleria de Agricultura of Generalitat Valenciana and were performed in accordance with guidelines of the Directive of the European Commission (2010/63/EU) for care and management of experimental animals. Experiments complied with the ARRIVE guide for animal experimentation. Rats were randomly distributed into four groups: control; control + rifaximin; CCl_4_ and CCl_4_ + rifaximin. Four different experiments with 8 rats per group were carried out, using a total of 32 rats per group. The number of rats used for each parameter is indicated in the corresponding figure legend. Rats were sacrificed at 4 weeks to analyze alterations in the hippocampus.

### 2.2. Immunohistochemistry

Rats were anaesthetized with sodium pentobarbital (1 mL/kg body weight) and transcardially perfused with 0.9% saline followed by 4% paraformaldehyde in 0.1 M phosphate buffer (pH 7.4). Brains were removed and post-fixed in the same fixative solution for 24 h at 4 °C. Five-micrometer thick, paraffin-embedded sections (5 µm) were cut and mounted on coated slide glass. Sections were rehydrated and antigen retrieved with the Dako 3 in 1 AR buffer EDTA pH 9.0 in a DAKO PT link and then processed with the Envision Flex kit (DAKO) blocking endogenous peroxidase activity for 5 min and then incubated with primary antibodies. Primary antibodies used for the study were: anti-ionised calcium binding adapter molecule 1 (IBA1) (Wako (019-19741); 1:300 for 30 min), anti- Glial Fibrillary Acidic Protein (GFAP) (Dako (IR524); ready to use for 20 min), anti-IL-1β (Abcam (9722; 1:300), anti-TNFα (Abcam (ab6671); 1:300), and CCL2 (Proteintech (66272); 1:200). To visualize the reaction Envision Flex + horseradish peroxidase was added for 20 min and finally diaminobenzidine (DAB) was added for 10 min. Mayer’s hematoxylin (DAKO S3309; Ready to use) for 5 min was used to nucli staining.

### 2.3. Analysis of IL-1β, TNFα and CCL2 Expression in the CA1-Region of Hippocampus

Analysis of these cytokines in the hippocampus was performed on 10 40×-fields per rat randomly photographed. The mean gray value in the pyramidal neurons of the CA1 layer was calculated using ImageJ software.

### 2.4. Analysis of Astrocytes and Microglia Activation

Sections stained with IBA1 and GFAP were scanned with Panoramic Scanner (3DHISTECH, Budapest, Hungary) and photographs were taken with Panoramic viewer software (3DHISTECH, Budapest, Hungary). Analysis of IBA1 and GFAP staining was performed in the whole hippocampus using the Image J software. To measure the perimeter of microglia in white matter of cerebellum, 2000–20,000 size filter was applied. For each rat at least 10 fields (56×) were photographed and 30–40 cells were quantified. The 10 fields were randomly chosen to cover most of the hippocampus. The results were then converted from pixels to micrometers. For GFAP analysis no size filter was applied. Ten fields (56×) for rat were randomly photographed and the total GFAP stained area was quantified. The results were expressed as percentage of GFAP stained area.

### 2.5. Membrane Surface Expression of Subunits of AMPA and NMDA Glutamate Receptors

It was analyzed as described in [42]. Rats were euthanized by decapitation and each dissected hippocampus was put into ice-cold Krebs buffer (in mmol/L): NaCl 119, KCl 2.5, KH_2_PO_4_ 1, NaHCO_3_ 26.2, CaCl_2_ 2.5, and glucose 11, aerated with 95% O_2_ and 5% CO_2_ at pH 7.4. Transversal 400 μm thick slices were obtained with a chopper. Slices were added to tubes containing ice-cold Krebs buffer with or without 2 mM bis(sulfosuccinimidyl)suberate (BS3) (Pierce, Rockford, IL, USA) and incubated for 30 min at 4 °C. Cross-linking was terminated by adding 100 mM glycine (10 min, 4 °C). The slices were homogenized by sonication for 20 s. Samples treated with or without BS3 were analyzed by Western blot using antibodies against NR1 (NMDA Receptor subunit 1) subunit of NMDA receptor (1:1000; BD Biosciences, Franklin Lakes, NJ, USA), NR2B (NMDA Receptor subunit 2B) subunit of NMDA receptor (1:1000; Millipore, MA, USA), NR2A (NMDA Receptor subunit 2A) subunit of NMDA receptor (1:1000; Millipore), GluA1 subunit of AMPA receptor (1:1000; Millipore) GluA2 subunit of AMPA receptor (1:2000; Millipore). The surface expression of receptor subunits was calculated as the difference between the intensity of the bands without BS3 (total protein) and with BS3 (non-membrane protein) [27].

### 2.6. Novel Object Recognition (NOR) and Novel Object Location (NOL) Memory Tests

Tests were performed in an open-field arena (70 × 70 × 40 cm) with visuospatial cues on the walls as in [19]. Habituation was performed during 5 days by allowing the rats freely explore the empty field arena for 5 min each day The NOL test was performed on day 6. First, two identical objects were placed in the arena. After freely exploring for 3 min the rat was put into its cage for two h. After that, the test was performed, moving one of the objects to a different location and allowing the rat to freely explore again for 3 min. The NOR test was performed on day 7. As in NOL, two identical objects were placed in the arena, and rats freely explored for 3 min. In this case the rat was put into its cage waiting 6 hours to perform the test, which consisted of changing one of the objects for an unexplored object and allowing the rat to freely explore again for 3 min. A discrimination ratio was calculated, considering individual differences in the total time of exploration. The discrimination ratio was calculated as the difference between the time spent exploring the object whose location had been changed (NOL) or the new, unexplored object (NOR) with the object that was still in its initial position.

### 2.7. Radial Maze

Radial maze was performed as in [16], in a maze consisting in 8 arms (70 cm long and 10 cm wide) radially distributed from a central area with a diameter of 30 cm. Rats were habituated during 2 sessions allowing them to freely explore the maze for 5 min each day (at first session pellets were located along all the maze while the second day pellets were placed at the end of all arms). Test was performed during 4 days (three trials per day). The task involved locating two pellets, each placed at the end of a different arm according to a random configuration. Configurations were specific for each rat and were kept invariable throughout training. The number of spatial reference errors (first visits to arms with no food) and working errors (visits to arms already visited in the same trial) were calculated. A learning index was calculated as the number of right choice–number of errors.

### 2.8. Statistical Analysis

GraphPad Prism software v. 7.0 was used to statistical analysis. Data are expressed as mean ± SEM. Statistical analysis was carried out using one-way ANOVA and Tukey’s multiple comparisons test or two-way ANOVA with repeated measures and Bonferroni’s multiple comparisons test, when appropriate. Data that did not pass the normality test (D’Agostino and Pearson or Komogorov–Smirnov tests) were analyzed with the nonparametric test Kruskal–Wallis test, with Dunn’s test for multiple comparisons. When standard deviations (SDs) were not equal, Welch’s ANOVA with Dunnett’s T3 multiple comparisons test was applied. A confidence level of 95% was considered as significant.

## 3. Results

### 3.1. Rats with Mild Liver Damage Show Microglia and Astrocyte Activation in the Hippocampus Rifaximin Reverses Microglia but Not Astrocyte Activation

**Figure 1:**
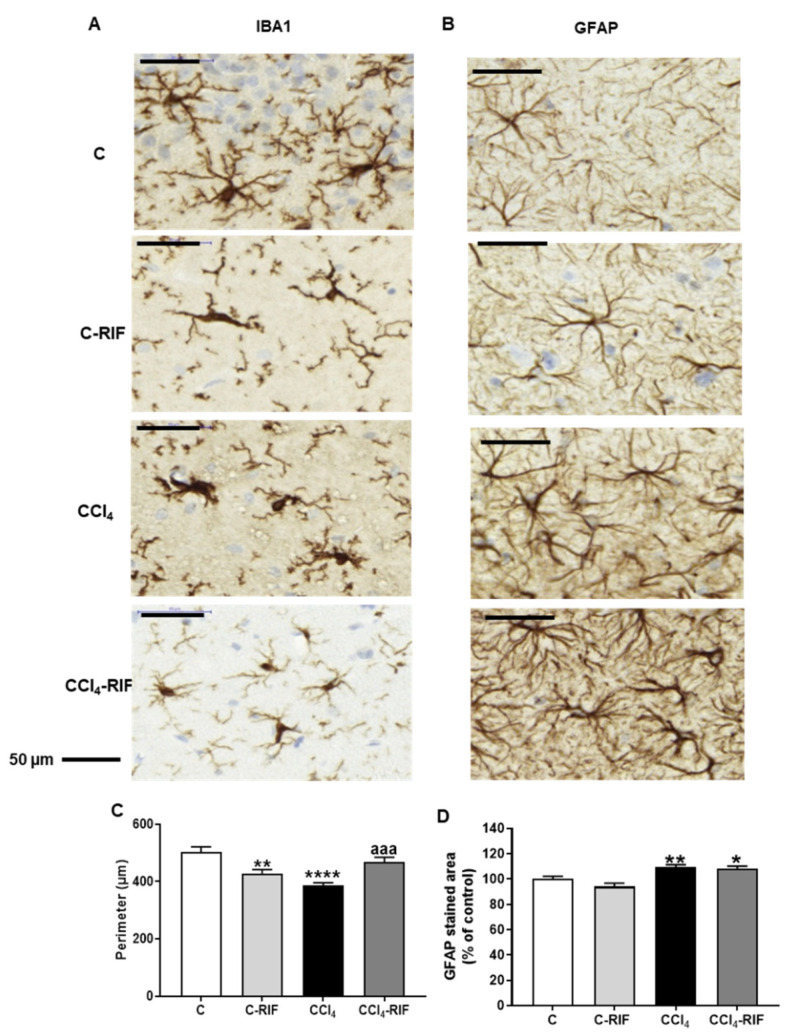
Astrocyte and microglia activation in the hippocampus. The perimeter of the microglia (stained with Iba1 marker (**A**)) and the percentage of GFAP stained area (**B**) was analyzed. Values are the mean ± SEM of 8 rats per group. In (**C**) the data were analyzed using the non-parametric Kruskal–Wallis test and Dunn’s multiple comparisons test. In (**D**) the data were analyzed using a one-way ANOVA and Tukey’s multiple comparisons test. The asterisks indicate a significant difference with respect to the control group * *p* < 0.05, ** *p* < 0.01, **** *p* < 0.0001, “aaa” *p* < 0.001. C: control; C-RIF: Control rats + rifaximin; CCl_4_: CCl_4_ injected rats and CCl_4_-RIF: CCl_4_ rats + rifaximin.

At four weeks of CCl_4_ injections, microglia were activated in the hippocampus (386 ± 11 µm for CCl_4_ rats compared to 504 ± 17 µm in controls, *p* < 0.0001). Treatment with rifaximin for two weeks reversed microglial activation in rats with mild liver failure (469 ± 16, *p* < 0.001) and induced microglial activation in control rats (428 ± 14 µm, *p* < 0.01) (Figure 1A,C).

Astrocytes activation was also observed in the hippocampus at 4 weeks of CCl_4_ injections. GFAP stained area increased (*p* < 0.01) in CCl_4_ rats to 110 ± 1.8% of control rats and remained at 108 ± 2.1% in CCl_4_ rats treated with rifaximin (*p* < 0.05 compared to controls), indicating that rifaximin treatment does not reverse astrocytes activation (Figure 1B,D).

**Figure 2:**
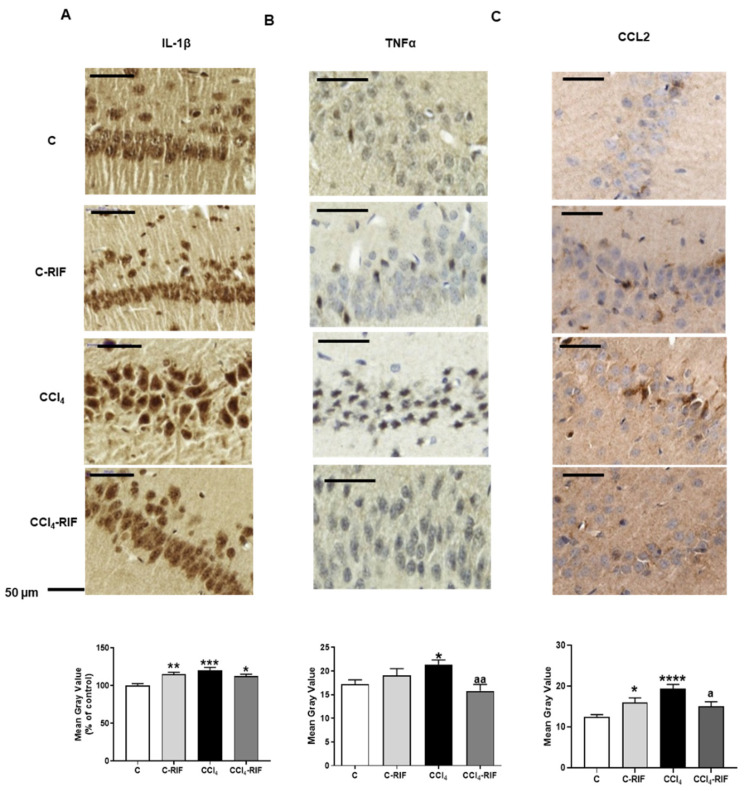
IL-1β (**A**), TNFα (**B**), and CCL2 (**C**) content in CA1 region of hippocampus. It was analyzed by immunohistochemistry. The quantification was expressed according to the mean on the grey scale (Mean Grey Value) and as percentage of control rats for IL-1β quantification. Values are the mean ± SEM of 6 rats per group. The data were analyzed using a one-way ANOVA and Tukey’s multiple comparisons test. The asterisks indicate a significant difference compared to the control group * *p* < 0.05, ** *p* < 0.01, *** *p* < 0.001, **** *p* < 0.0001; “a” compared to the CCl_4_ rats “a” *p* < 0.05, “aa” *p* < 0.01.

We also analyzed by immunohistochemistry the content of IL-1β, TNFα, and CCL2 in the neurons of the CA1 region of hippocampus. IL-1β levels were increased in rats with mild liver damage (120 ± 4% of controls, *p* < 0.001), in control rats treated with rifaximin (115 ± 3%, *p* < 0.01), and in the CCl_4_ rats treated with rifaximin (113 ± 2%, *p* < 0.05), indicating that rifaximin does not prevent the increase in IL-1β (Figure 2A).

TNFα levels were increased in CA1 neurons of CCl_4_ injected rats (21 ± 1 a.u.) compared to control rats (17 ± 1 a.u., *p* < 0.05). Rifaximin reversed the increase in TNFα (15.8 ± 1.4 a.u.) in CCl_4_ rats (*p* < 0.01; Figure 2B).

CCL2 content increased (*p* < 0.0001) in the CA1 neurons in CCl_4_ rats (19 ± 1 a.u.) compared to 12 ± 0.6 a.u. in controls. Rifaximin treatment reduced this increase (15 ± 1 a.u., *p* < 0.05) in CCl_4_ rats and increased CCL2 content in control rats (16 ± 1 a.u. *p* < 0.05) (Figure 2C).

### 3.2. Hippocampus of Rats with Mild Liver Damage Shows an Increase in Infiltrated Macrophages and Lumphocytes

**Figure 3:**
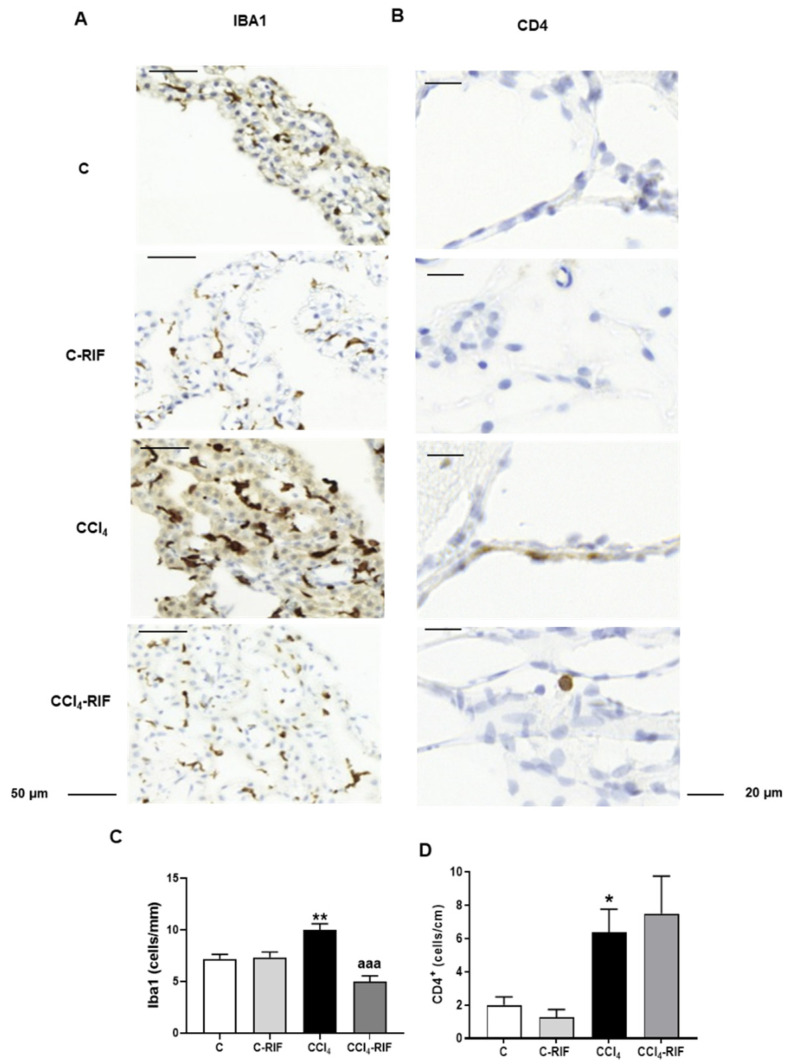
Infiltration of macrophages (**A**) and CD4+ T lymphocytes (**B**) into meninges of the hippocampus. It was analyzed by immunohistochemistry with anti-Iba1 or anti-CD4 markers, respectively. Values are the mean ± SEM of 5 rats per group. In (**C**) data were analyzed using one-way ANOVA and Tukey’s multiple comparisons test and in (**D**) using a Welch’s ANOVA test and Dunnett’s multiple comparisons test. The asterisks indicate a significant difference compared to the control group * *p* < 0.05, ** *p* < 0.01, and “aaa” *p* < 0.001.

As CCL2 promotes monocytes and lymphocytes infiltration from blood, we analyzed in the meninges of hippocampus the presence of macrophages (marked with Iba1) or lymphocytes (marked with CD4). Rats with mild liver damage showed increased number of both, macrophages (10.0 ± 0.6 cells in CCl_4_, compared to 7.2 ± 0.4 cells in control rats, *p* < 0.01), and lymphocytes (6.4 ± 1.4 cells in CCl_4_ rats, compared to 2.0 ± 0.5 cells in control rats, *p* < 0.05) in the meninges of the hippocampus (Figure 3A–D). Treatment with rifaximin completely prevented the increase in macrophages (5.0 ± 0.6 cells in CCl_4_-RIF, *p* < 0.001 vs. CCl_4_ group) but not that of lymphocytes (6.9 ± 2.6 in CCl_4_-RIF group) (Figure 3A–D).

### 3.3. Membrane Expression of NMDA and AMPA Receptor Subunits Is Altered in the Hippocampus of Rats with Mild Liver Damage

**Figure 4:**
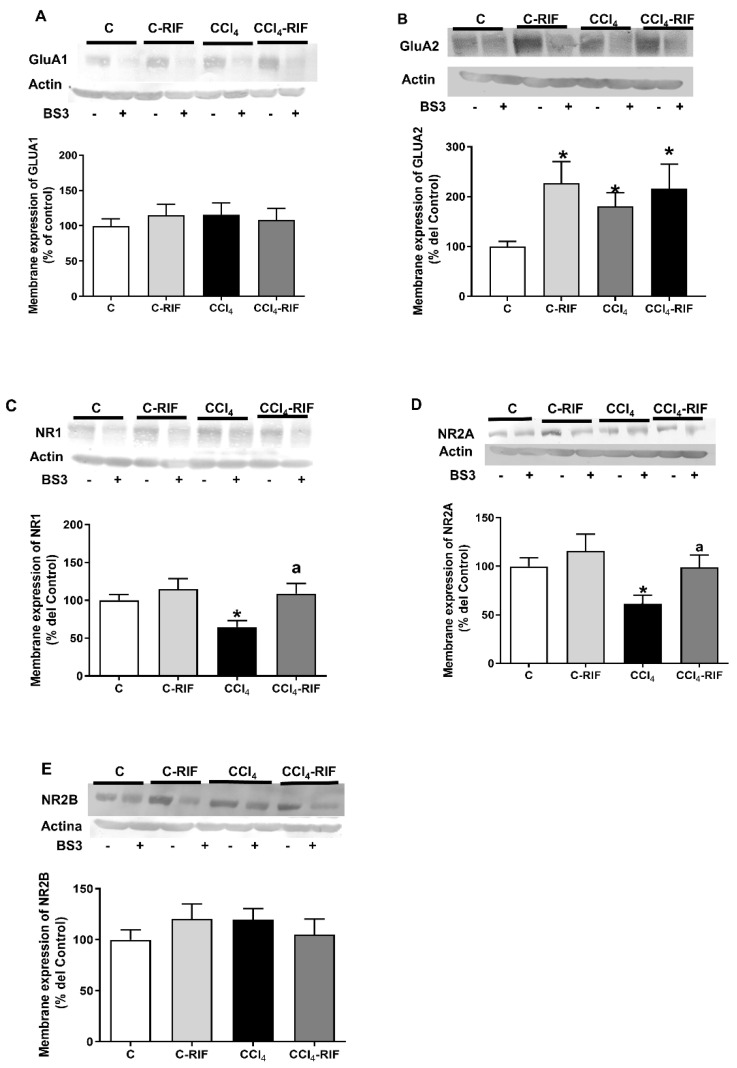
Membrane expression of GluA1 (**A**) and GluA2 (**B**) AMPA receptor subunits; of NR1 (**C**), NR2A (**D**) and NR2B (**E**) NMDA receptor subunits in the hippocampus. Samples from slices with (+) or without (-) the crosslinker BS3 were analyzed in the same western blot and the surface expression of receptor subunits was calculated as the difference between the intensity of the bands without BS3 (total protein) and with BS3 (non-membrane protein). Values are the mean ± SEM of 15 rats per group. Data were analyzed using a one-way ANOVA and Tukey’s multiple comparisons test except for GluA2 (**B**) that Welch’s ANOVA test and Dunnett’s multiple comparisons test was used. The asterisks indicate a significant difference with respect to the control group * *p* < 0.05. The “a” indicate a significant difference with respect to the CCl_4_ rats “a” *p* < 0.05.

Membrane expression of GluA1 was not altered by CCl_4_ or rifaximin (Figure 4A). Rats with mild liver damage show increased membrane expression of the GluA2 subunit of AMPA receptors (181 ± 27% of controls, *p* < 0.05). Membrane expression of GluA2 was also increased in both control and CCl_4_ rats treated by rifaximin (227 ± 43%, *p* < 0.05 in controls and 216 ± 49%, *p* < 0.05 in CCl_4_ rats) (Figure 4B).

Regarding NMDA receptor, the membrane expression of NR1 and NR2A subunits was reduced in rats with mild liver damage compared to control rats (65 ± 8%, *p* < 0.05 and 61 ± 9%, *p* < 0.05, respectively; Figure 4C,D), whereas membrane expression of the NR2B subunit was not significantly affected (Figure 4E). Treatment with rifaximin normalized membrane expression of NR1 and NR2A subunits in rats with mild liver damage (109 ± 13%, *p* < 0.05 and 99 ± 13%, *p* < 0.05) (Figure 4C,D). Rifaximin did not affect membrane expression of NMDA receptor subunits in control rats.

### 3.4. Spatial Learning and Memory Are Impaired in Rats with Mild Liver Damage While Non-Spatial Memory and Working Memory Are Not Altered

**Figure 5:**
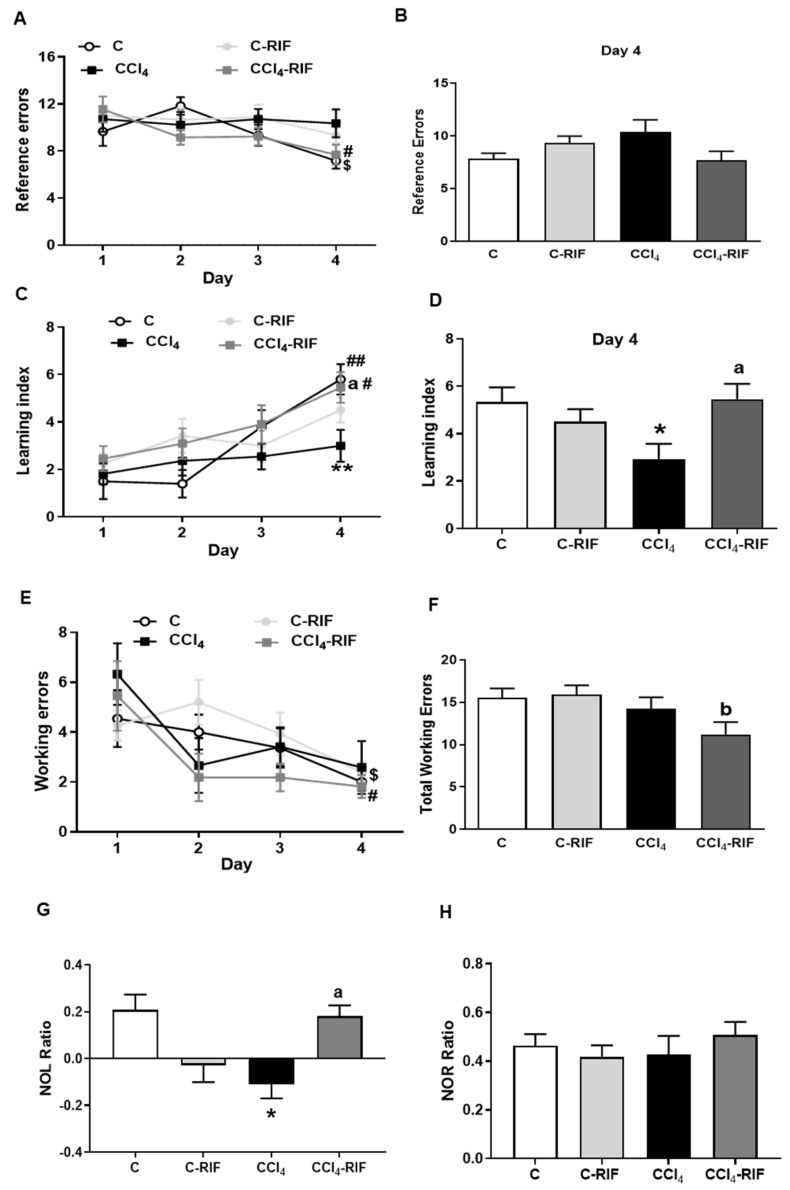
Spatial learning and reference and working memory in the radial maze and novel object location (NOL) and recognition (NOR) memory tests. Reference memory (**A**,**B**) spatial learning (**C**,**D**), and working memory (**E**,**F**) were evaluated in the radial maze. Values are the mean ± SEM of 15 rats per group. In (**A**,**C**,**E**) the data were analyzed using a two-way ANOVA with repeated measures and Tukey’s multiple comparison test. In (**A**), only for time a significant effect was found (F (2.856, 142.8) = 4.332, *p* < 0.01); in (**C**)the effect between groups was significant (F (3, 40) = 2.925, *p* < 0.05) as well as the time effect (F (2.949, 118.0) = 13.69, *p* < 0.0001); in (**E**), only the time was significant (F (3, 72) = 4.24, *p* < 0.01). In B and F non-parametric Kruskal–Wallis test and Dunn’s multiple comparisons test was used and no significant effects were found. In (**D**) one-way ANOVA and Tukey’s multiple comparisons test was used and statistic value was F (3, 45) = 3.789, *p* < 0.05. In multiple comparisons, the asterisks indicate a significant difference with respect to the control group * *p* < 0.05, ** *p* < 0.01, “a” with respect to CCl_4_ rats “a” *p* < 0.05 and “b” compared with C-RIF group, “b”, *p* < 0.05. The symbol # indicates within the same group significant difference with respect to day 1 “#” *p* < 0.05 and “##” *p* < 0.01. The $ symbol indicates within the same group a significant difference with respect to day 2 “$” *p* < 0.05. Rats were evaluated for the ability to identify the change in location of an object through the NOL test (**G**) and to identify a new object by the NOR test (**H**). Values are mean ± SEM of 13 rats per group. Data were analyzed using a one-way ANOVA and Tukey’s multiple comparison test (F(3, 34) = 5.389 for NOL test). The asterisks indicate a significant difference with respect to the control group * *p* < 0.05, and “a” with respect to the CCl_4_ rats “a” *p* < 0.05.

We then analyzed if hippocampal neuroinflammation and alterations in glutamate receptors induce cognitive impairment in rats with mild liver failure. Spatial learning and working memory were analyzed using the radial maze test. Reference errors were not significantly affected in CCl_4_ rats compared to control rats (Figure 5A,B). However, rifaximin increased slightly reference errors in control rats, suggesting a mild learning impairing (Figure 5A). At day 4 there was a tendency to increase the reference errors in CCl_4_ rats compared with controls, but the difference did not reach statistical significance (Figure 5B).

The learning index increased over the 4 days of test in all groups except for CCl_4_ rats, which do not improve learning (Figure 5C). The learning index of rats with mild liver damage was lower than for control rats at day 4 (3.0 ± 0.67, *p* < 0.05, compared to 5.8 ± 0.65 in control rats; Figure 5D). Rifaximin treatment restored learning index in CCl_4_ injected rats at day 4 (5.5 ± 0.65, *p* < 0.05; Figure 5D).

Working errors decreased along days of training, similarly in all groups. No significant differences in total working errors were observed between different groups, except for a lower number of total working errors on the fourth day of the test in CCl_4_ rats treated with rifaximin (Figure 5E,F).

We also analyzed novel object location (NOL test) as a measure of spatial memory and novel object recognition memory (NOR test) as a non-spatial memory.

CCl_4_ rats showed a lower discrimination ratio compared to control rats in the novel object location (−0.11 ± 0.06 vs. 0.21 ± 0.06 in controls, *p* < 0.05). Treatment with rifaximin restores object location memory in rats with mild liver damage (0.18 ± 0.05, *p* < 0.05) and tended to reduce location memory in control rats, but the effect was not significant (Figure 5G).

NOR test was also performed at three weeks to evaluate non-spatial memory. Rats with mild liver damage did not show impaired object recognition memory (Figure 5H) and rifaximin did not affect it either.

## 4. Discussion

The results reported (summarized in Table 1) show that rats with mild liver damage, steatosis and hepatic inflammation, with incipient fibrosis, similar to steatohepatitis in human patients, already show neuroinflammation in the hippocampus. This is associated with altered glutamatergic neurotransmission, with altered membrane expression of AMPA and NMDA receptors subunits, and impaired spatial learning and memory, indicating that this mild liver damage already induces hippocampus-dependent neurological impairment.

Patients with mild steatohepatitis would also present neuroinflammation in the hippocampus, as reported in the cerebellum [21], which would contribute to mild cognitive impairment reported in these patients [2,3,4], and our results in rats with mild liver damage in this study support this and describe some potential underlying mechanisms. Up to 32% of patients diagnosed with NAFLD have mild cognitive impairment. This study supports the therapeutic use of rifaximin also in patients with NAFLD, not only in cirrhotics, presenting cognitive impairment.

Rats with mild liver damage show infiltration of monocytes (macrophages) and CD4+ lymphocytes in the hippocampus. This is associated with increased levels of CCL2 in neurons and with activation of microglia and astrocytes. IL-1β and TNFα levels are also increased in neurons in the CA1 region of hippocampus and this is associated with increased membrane expression of the GluA2 subunit of AMPA receptors and reduced membrane expression of the NR1 and NR2A subunits of NMDA receptors. The altered membrane expression of glutamate receptors is also associated with impaired spatial learning and memory, reflected in reduced learning index in the radial maze and impaired novel object location memory.

**Figure 6:**
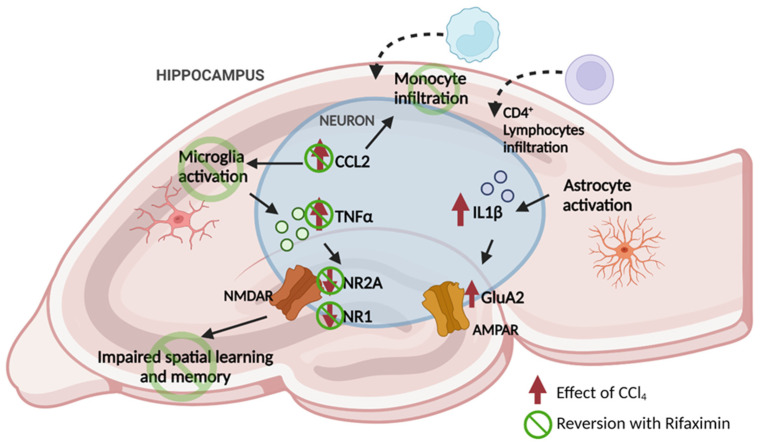
Proposed mechanisms leading to the impaired learning and memory in rats with mild liver damage. These results suggest that the increase in CCL2 in the hippocampus would be an early event in the process leading to cognitive impairment in rats with mild liver damage. The increase in CCl2 would promote both the infiltration of monocytes into the hippocampus to became macrophages and the activation of microglia. Microglia activation in turn would contribute to the increase in TNFα in hippocampal neurons of these rats, leading to the reduced membrane expression of NR1 and NR2A subunits of NMDA receptors which, in turn, would lead to the impairment in spatial learning and memory. All this process is reversed by treatment with rifaximin, that restores cognitive function in rats with mild liver damage. Mild liver damage also triggers infiltration of CD4+ lymphocytes, activation of astrocytes, increased levels of IL-1β and enhanced membrane expression of the GluA2 subunit of AMPA receptors in the hippocampus. These changes are not reversed by treatment with rifaximin. Created with BioRender.com.

Treatment with rifaximin reverses the alterations in CCL2, in monocytes infiltration and microglia activation, in TNFα in neurons, in membrane expression of NR1 and NR2A and in spatial learning and memory. The fact that treatment with rifaximin reverses all these steps support that the process, summarized in Figure 6, would be the main mechanism by which mild liver damage impairs spatial learning and memory and rifaximin restores them. However, rifaximin treatment does not reverse the infiltration of CD4+ lymphocytes, activation of astrocytes and the increase in IL-1β and in membrane expression of GluA2, suggesting that these effects contribute to a lesser extent to spatial memory impairment in our rats.

These results suggest that the increase in CCL2 in the hippocampus would be an early event in the process leading to cognitive impairment in rats with mild liver damage as summarized in Figure 6. The increase in CCl2 would promote both the infiltration of monocytes into the hippocampus to became macrophages and the activation of microglia.

This proposed mechanism is supported by reports in the literature. Infiltration of peripheral monocytes/macrophages is promoted by CCL2 and chemokine (C-X3-C motif) ligand 1 (CX3CL1 or fractalkine) [43,44]. We show here that CCL2 is increased in the hippocampus of rats with mild liver damage. Moreover, we previously showed that these rats have increased plasma levels of CX3CL1 at 4 weeks of CCl_4_ treatment [36]. This suggest that the increased levels of CCL2 in the hippocampus and of CX3CL1 in blood of these rats would contribute to the infiltration of monocytes in the hippocampus.

It has been reported that CCL2 promotes microglia activation through activation of its receptor CCR2 [45,46,47]. The increase in CCL2 in rats with mild liver damage would also contribute to microglia activation in the hippocampus.

Microglia activation in turn would contribute to the increase in TNFα in hippocampal neurons of these rats, as occurs in rats with chronic hyperammonemia and hepatic encephalopathy [15,16,17,20,24,48,49,50], in meningitis [51], in dorsal horn neurons and the spinal cord [52,53], or in propofol-induced neurotoxicity [54].

The increased levels of TNFα in turn would contribute to the reduced membrane expression of the NR2A subunits of NMDA receptors in the hippocampus of rats with mild liver damage. Balosso et al. [55] reported that in TNFR2 (p75) knockout mice membrane expression of NR2A/B in the hippocampus is increased, suggesting that activation of TNFR2 by TNFa shold lead to a decrease in membrane expression of these subunits as we find in the present study.

It has been reported that both TNFα and IL-1β may modulate membrane expression of the NR1, NR2A, and NR2B subunits of NMDA receptors and of the GluA1 and GluA2 subunits. However, the effects reported are different depending on the experimental conditions, the pathology studied or the concentration of TNFα and IL-1β. An opposite effect of TNFα and IL-1β on GluA1 membrane expression has been reported. TNFα selectively enhances membrane expression of GluA1 in hippocampal neurons [56]. In contrast, IL-1β increased membrane expression of GluA1 more slightly [57] or, at high concentrations, reduces it [58]. An increase in membrane expression of GluA1 induced by TNFα has been also reported in [18,59,60,61] while a decrease in membrane expression of GluA1 induced by IL-1β has been reported in [19,55,62,63,64,65].

Opposite effects of TNFα and IL-1β on membrane expression of NR2A and NR2B have also been reported in [18,66,67].

This suggests that the final effect on membrane expression of GluA1, NR2B, and NR2A would depend on the grade of neuroinflammation and on the total and relative concentrations of TNFα and IL-1β. It is likely that a similar differential modulation occurs for NR1 and GluA2.

In rats with chronic hyperammonemia and additional neuroinflammation due to insertion of canula into the cerebral ventricle, Cabrera-Pastor et al. [18] propose that increased levels of TNFα enhance membrane expression of GluA1 and reduces that of GluA2 while increased levels of IL-1β increase membrane expression of NR1 and NR2A. However, Taoro-González et al. [62] showed that, in hippocampal slices from hyperammonemic rats (without the intracerebral canula) increased levels of IL-1β are responsible for the increased membrane expression of NR2B and GluA2 and for reduced membrane expression of GluA1.

In the rats with mild liver damage used in the present work the increased levels of TNFα are associated to a reduced membrane expression of the NR1 and NR2A subunits of NMDA receptors in the hippocampus and the changes in both TNFα and NR1 and NR2A are reversed by treatment with rifaximin.

Rats with mild liver damage show impaired spatial learning and memory while non-spatial memory was not impaired. This suggests that impaired spatial learning and memory is an early event in the progression of liver disease and that working and recognition memory would be impaired at more advanced stages of liver damage, when there is also hyperammonemia [36]. Rats with chronic hyperammonemia show impaired working and novel recognition memory [18,19], suggesting that peripheral hyperammonemia would be a main contributor to this type of cognitive impairment while other factors would mediate impairment of spatial learning and memory.

Different mechanisms for these two types of neuroinflammation-induced memory impairments have been already reported in hyperammonemic rats by Taoro-Gonzalez et al. [19], supporting a role for neuroinflammation in other brain areas, such as prelimbic and post and perirhinal cortex. A differential role of hippocampus in location and recognition memory has been reported [10], suggesting that spatial memory performance involves other brain areas in addition to hippocampus, while recognition memory would not. These differential underlying mechanisms would explain why spatial memory, but not recognition memory, is impaired in our model of mild liver damage.

The role of glutamate receptors on spatial learning and memory has been well established. The literature reports show that both increase or decrease in hippocampal expression of the different NMDA receptor subunits can induce learning and memory impairments [68,69,70,71,72], and underly the importance of the relation between NR2A and NR2B subunit content [73,74,75]. We show here that mild liver damage is associated to decreased membrane expression of NMDA subunits NR1 and NR2A with not change in NR2B subunit, indicating a decrease in NRA/NR2B ratio. Louveau et al. [76] reported that mice lacking the protein CD3ζ show reduced postsynaptic localization of NR2A and spatial learning and memory deficits in the Barnes and NOL tests, supporting the contribution of NR2A to these spatial learning and memory tasks also in rats with mild liver damage.

Moreover, membrane expression of AMPA subunit GluA2 was increased in CCl_4_ rats, as well as in control or CCl_4_ rats treated with rifaximin, suggesting a minor role in the induction of spatial learning impairment, as this was not affected by rifaximin in control rats. However, increased GluA2 could participate in object location memory, partially impaired by rifaximin in control rats. The decrease in NR1 and increase in GluA2 were also reported [17] in rats with porta-cava anastomosis (PCS), another widely used animal model of liver failure and HE. Decreased membrane expression of AMPA and NMDA receptor subunits is involved in impaired long-term potentiation in the hippocampus of PCS rats [77], then impairing spatial learning and memory in the Morris water maze.

In addition to the CCL2–monocytes–microglia–TNFα–NMDA receptor–spatial learning and memory pathway summarized in Figure 6, mild liver damage also triggers infiltration of CD4+ lymphocytes, activation of astrocytes, increased levels of IL-1βand enhanced membrane expression of the GluA2 subunit of AMPA receptors (Figure 6). These changes are not reversed by treatment with rifaximin. This suggests that the mechanisms modulating the changes in these parameters are different to those discussed above. The infiltration of CD4+ lymphocytes and the activation of astrocytes would lead to the increase in IL-1βlevels, which in turn would increase membrane expression of GluA2, as suggested by Taoro-Gonzalez et al. [62] for hyperammonemic rats.

An important contribution of endotoxemia, that is, of the increase in LPS, to appearance of NAFLD has been described [78]. On the other hand, the induction of neuroinflammation and cognitive alterations due to LPS has also been described, mainly in studies on the mechanisms of Alzheimer’s disease [79,80]. Therefore, we cannot exclude the contribution of this endotoxin to the appearance of neurological alterations in NAFLD. Furthermore, the model we have used in this study, CCl_4_-induced liver damage, has been reported to induce an increase in LPS [81,82]. The main mechanism of LPS-induced damage is the induction of oxidative stress. Oxidative stress also contributes to the development of liver damage and is present in CCl_4_-induced liver injury [78,83]. In addition, oxidative stress, associated with neuroinflammation, also contributes to cognitive alterations in different pathologies [84,85], including MHE [16,86,87,88,89], and therefore could play a role in the impaired spatial memory in our model.

Future research would be necessary to delve into the role of endotoxemia and oxidative stress in the appearance of neurological alterations and MHE in NAFLD.

Rifaximin has been shown to decrease endotoxemia in cirrhotic patients [90,91,92,93,94] and also in animal models of cirrhosis [95,96]. However, its effect on endotoxemia in NAFLD or in patients or animal models with mild liver injury in general has not been well studied, and yet few studies have been conducted on the effect of rifaximin on NAFLD in patients [97,98,99] or in animal models [100,101]. Although a main mediator of the damage produced by LPS is oxidative stress, this has not been analyzed in any of these studies. Therefore, future studies should analyze the effect of rifaximin on endotoxemia and oxidative stress in NAFLD.

## 5. Conclusions

These results suggest that the increase in CCL2 in the hippocampus would be an early event in the process leading to cognitive impairment in rats with mild liver damage. The increase in CCl2 would promote both the infiltration of monocytes into the hippocampus to became macrophages and the activation of microglia. Microglia activation in turn would contribute to the increase in TNFα in hippocampal neurons of these rats, leading to the reduced membrane expression of NR1 and NR2A subunits of NMDA receptors which, in turn, would lead to the impairment in spatial learning and memory. All this process is reversed by treatment with rifaximin, that restores cognitive function in rats with mild liver damage. Mild liver damage also triggers infiltration of CD4+ lymphocytes, activation of astrocytes, increased levels of IL-1β and enhanced membrane expression of the GluA2 subunit of AMPA receptors in the hippocampus. These changes are not reversed by treatment with rifaximin. These results show that mild liver damage, such as that present in NAFLD, already induces neuroinflammation and cognitive alterations and that rifaximin treatment reduces neuroinflammation and improves cognitive impairment in these rats, suggesting that rifaximin could be also used as treatment to improve mild cognitive impairment in NAFLD patients.

## Figures and Tables

**Table 1:** Summary of CCl_4_ and rifaximin effects.

	CCL_4_	C-RIF	CCL_4_-RIF
Microglia activation	↑	↑	Normalized
Astrocyte activation	↑	Not affected	↑
Content of IL-1β	↑	↑	↑
Content of TNFα	↑	Not affected	Normalized
Content of CCL2	↑	↑	Normalized
Macrophages in meninges	↑	Not affected	Normalized
CD4^+^ Lymphocytes in meninges	↑	Not affected	↑
Membrane expression of GluA1	Not affected	Not affected	Not affected
Membrane expression of GluA2	↑	↑	↑
Membrane expression of NR1	↓	Not affected	Normalized
Membrane expression of NR2A	↓	Not affected	Normalized
Membrane expression of NR2B	Not affected	Not affected	Not affected
Reference Errors	Not affected	Not affected	Not affected
Working Errors	Not affected	Not affected	Not affected
Learning Index	↓	Not affected	Normalized
NOL	↓	Not affected	Normalized
NOR	Not affected	Not affected	Not affected

The effects of CCl_4_-induced mild liver damage and those of rifaximin administration, both in control rats and in rats with liver damage, for each outcome analyzed (first column), are summarized. ↑ means increased and ↓ decreased. Normalized means that rifaximin normalize the alteration induced by CCl_4_.

## Data Availability

Data is contained within the article.
